# g-C_3_N_4_/CeO_2_ Binary Composite Prepared and Its Application in Automobile Exhaust Degradation

**DOI:** 10.3390/ma13061274

**Published:** 2020-03-11

**Authors:** Shengchao Cui, Baowen Xie, Rui Li, Jianzhong Pei, Yefei Tian, Jiupeng Zhang, Xiangyang Xing

**Affiliations:** 1 Highway school, Chang’an University, Xi’an 710064, China; cuishengchao@chd.edu.cn (S.C.); xiebaowen@chd.edu.cn (B.X.); lirui@chd.edu.cn (R.L.); zhjiupeng@163.com (J.Z.); xiangyang.xing@chd.edu.cn (X.X.); 2 School of Materials, Chang’an University, Xi’an 710064, China; yftian@chd.edu.cn

**Keywords:** g-C_3_N_4_/CeO_2_, exhaust gas, degradation efficiency, photocatalytic material, heterojunctions

## Abstract

Vehicle exhaust seriously pollutes urban air and harms human health. Photocatalytic technology can effectively degrade automobile exhaust. This work prepared g-C_3_N_4_/CeO_2_ photocatalytic material by constructing heterojunctions. Four kinds of g-C_3_N_4_/CeO_2_ composite photocatalytic materials with different mass ratios were prepared. An indoor exhaust gas purification test was carried out under natural light and ultraviolet light irradiations. The optimum mass ratio of g-C_3_N_4_ material and CeO_2_ material was determined by evaluating the exhaust gas degradation effective. Moreover, the structure and morphology of the g-C_3_N_4_/CeO_2_ composite were investigated with microscopic characterization experiments (including XRD, TG-DSC, FT-IR, UV-Vis, SEM and XPS). The results obtained were that the optimum mass ratio of g-C_3_N_4_ material to CeO_2_ material was 0.75. The degradation efficiencies under ultraviolet irradiation in 60 min for HC, CO, CO_2_, NO_X_ were 7.59%, 12.10%, 8.25% and 36.82%, respectively. Under visible light conditions, the degradation efficiency in 60 min for HC, CO, CO_2_ and NO_X_ were 15.88%, 16.22%, 10.45% and 40.58%, respectively. This work is useful for purifying automobile exhaust in the future.

## 1. Introduction

While the development of science and technology has created a lot of wealth for human society, it also consumes a lot of energy and emits a large amount of waste gas and water, thus causing serious environment pollution and endangering humans’ health [1]. Automobiles are the main contributors to atmospheric pollutant emissions, with CO, NO_X_ and PM emissions exceeding 90% and HC exceeding 80%. The main components contained in automobile exhaust are NO_X_, HC, CO and CO_2_. Nitrogen oxides can cause environmental problems such as acid rain and ozone depletion. Nitrogen oxides can also produce photochemical smog with HC. The most famous photochemical smog events are the London photochemical smog and Los Angeles photochemical smog events [2]. Carbon dioxide is a greenhouse gas that can raise the Earth’s temperature. Severe environmental pollution is destroying our ecosystem, so it is a significant task to govern and protect the environment [3].

In the 1970s, Japanese scholars Honda and Fujishma first found the photolysis of water under the bias condition by using a single crystal TiO_2_ photoelectrode [4], and they successfully decomposed water into H_2_ and O_2_. It was later found that the TiO_2_ material irradiated by light could not only decompose water but also be used to degrade organic pollutants. TiO_2_ material could deeply oxidize most of the toxic organic substances [5]. This major discovery attracted a large amount of scholars’ interests, and there have been many outcomes related TiO_2_ material [6,7,8,9,10,11,12]. However, TiO_2_ has a large forbidden band width (about 3.2 eV) and cannot absorb solar light efficiently. It can only absorb 4%–6% of the ultraviolet band energy in the solar spectrum. Though the modified TiO_2_ catalytic material has a greater performance than that of pure TiO_2_ material, the catalytic effect still cannot effectively break through. Therefore, it is very meaningful to find and study materials that can make full use of solar energy.

Graphite phase carbon nitride is a non-metallic semiconductor material with the advantages of non-toxicity, low cost, and easy preparation. It is widely used in the production of clean renewable energy and environmental pollution control technologies. Its forbidden band width is about 2.7 eV, which can directly use solar energy, thus attracting a lot of scholars’ attention and research [13,14,15,16,17,18]. However, the photogenerated electron-hole pairs of g-C_3_N_4_ are easily complexed and have a small specific surface area, resulting in a low photocatalytic activity [19,20]. Therefore, the photocatalytic activity of g-C_3_N_4_ could be improved by modifying methods. Building heterojunction is a common method for photocatalysts. For example, the photocatalytic efficiency of g-C_3_N_4_/BiVO_4_ heterojunction is almost 5.3 times higher than that of an individual g-C_3_N_4_ sample [21]. In addition, WO_3_/g-C_3_N_4_ heterostructure nanocomposites were found to possess excellent photocatalytic activity for degrading methyl orange (MO) and tetracycline (TC) under visible light irradiation [22]. In this work, CeO_2_ was introduced to improve the photocatalytic efficiency of g-C_3_N_4_.

The cerium oxide material is a good catalyst with good oxygen storage and redox properties. It is a cubic fluorite-type structure that is composed of a face centered cubic cation and an anion occupying the octahedral gap position [23]. In this structure, the cation of each cerium element coordinates with the eight nearest oxygen element anions, and each oxygen element anion coordinates with the four nearest cerium element cations [24]. CeO_2_ has a variable metal ion pair (Ce^3+^/Ce^4+^) under different redox reaction atmospheres. This property makes the CeO_2_ material have an excellent oxygen storage and release capacity [25,26,27]. Furthermore, CeO_2_ has a valence band of 2.35 eV and a conduction band of −0.23 eV, and it can form a composite heterojunction with graphite phase carbon nitride. The heterojunction structure can promote the dissociation of excitons and improve the separation efficiency of the photo-generated carriers of the catalyst, thereby achieving the purpose of improving the activity of the catalyst [28].

However, few works have focused on g-C_3_N_4_/CeO_2_ heterostructure nanocomposites. Therefore, this work prepared a g-C_3_N_4_/CeO_2_ heterostructure composite photocatalytic material with different mass ratios. An exhaust gas degradation test was carried out to obtain the optimal mass ratio of g-C_3_N_4_ and CeO_2_. Under the optimal preparation conditions, the exhaust gas degradation test was conducted in 60 min. Moreover XRD, TG-DSC, FT-IR, UV-Vis, SEM, and XPS were executed to verify the performance of the g-C_3_N_4_/CeO_2_ material.

## 2. Experiment and Methods

### 2.1. Materials and Equipment

The raw materials and test equipment are shown in Table 1 and Table 2, respectively.

### 2.2. Preparation

The steps for preparing g-C_3_N_4_/CeO_2_ are as follows:

(1) g-C_3_N_4_ was prepared by dicyandiamide thermal polymerization at 600 °C.

(2) Three grams of g-C_3_N_4_ powders were weighed and dispersed it into a mixed solvent of 10 mL of deionized water and 40 mL of absolute ethanol. The mixed solution was stirred evenly and recorded as solution A. Then, 3 g of CeO_2_ powders were weighed and added to solution A, stirred, and recorded as solution B.

(3) A magnetic stirrer was used to stir solution B for 2 h, and then solution B was put in an oven at 80 °C for 12 h. The block solid could be obtained. Then, it was ground into powder and placed in a ceramic crucible.

(4) The crucible was placed in a muffle furnace at a heating rate of 15 °C/min and heated to 500 °C for 4 h, and then it was cooled down to 450 °C for 12 h. The sample that was obtained after calcination and cooling was the g-C_3_N_4_/CeO_2_ binary composite material.

(5) According to the above method, binary composite materials with 1:0.5, 1:0.75, 1:1 and 1:2 mass ratios of g-C_3_N_4_ to CeO_2_ were prepared.

### 2.3. Indoor Exhaust Gas Purification Step and Evaluation Method

Purification efficiency (η) can be used to evaluate the purification effect of the prepared materials on the exhaust gas. The purification efficiency formula can be written as the follows:

(1) $$\eta =\frac{C_{0}-C_{t}}{C_{0}}\times 100\%$$

where η is the purification efficiency, C_0_ is the initial exhaust concentration, and C_t_ is the exhaust concentration after t minutes.

**Figure 1:**
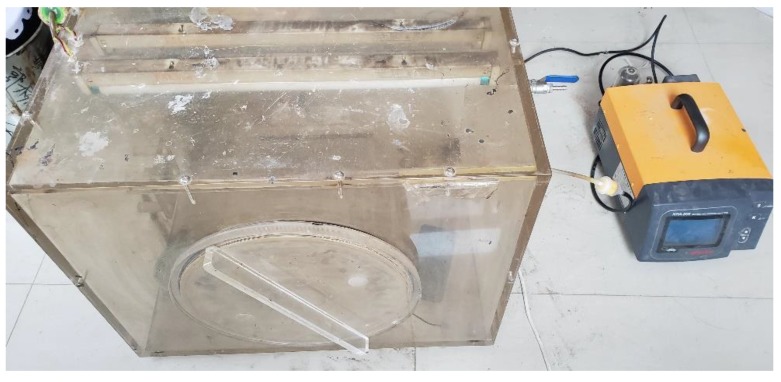
The exhaust purification system [29].

Therefore, we proposed a method for indoor exhaust gas purification. The indoor test process was as follows:

(1) The g-C_3_N_4_/CeO_2_ photocatalytic material was prepared by following the above steps, and 3 g of g-C_3_N_4_/CeO_2_ were weighed and then spread evenly on clean A4 paper;
(2) The exhaust purification system was connected in sequence. The exhaust purification system in this work is shown in Figure 1. Firstly, the DTN220B-NO_2_ portable nitrogen dioxide detector was put into the reaction box, and a small fan was turned on to keep gas flowing. Then, the photocatalytic material was put into the reaction box, which was closed tightly. Secondly, the reaction chamber was covered with a curtain to prevent light from entering. Then, the air inlet valve was opened, and the air outlet valve was closed. The engine was connected to the air inlet and injected exhaust gas into the reaction box.
(3) The NHA-506 (5G) car exhaust gas analyzer (Nanhua Instrument Co., Ltd., Foshan, China) was turned on. After injecting for a period of time, the automobile exhaust gas analyzer was used to test the concentration of various components in the exhaust gas. When a certain concentration was reached, the intake valve was closed and the gasoline engine was turned off. The UV or incandescent lamp was turned on and the test was started.
(4) The concentrations of NO, NO_2_, CO, CO_2_ and HC were recorded every 10 min. A total of 60 min and 7 groups of data were recorded.

### 2.4. Error Correction of Exhaust Gas Purification Test

Because the engine parameters of each car are different, the exhaust gas concentrations that are produced are also different. In this work, it was concluded that each component was in a certain range after a large number of repeated works. Through a large number of inflation tests, it could be found that the concentration range of HC was 340–400 ppm, CO was 4%–6%, CO_2_ was 5%–9%, and NO_X_ was 40–80 ppm. The principle of this tail gas purification experiment was to use a car tail gas analyzer to suck the tail gas out of the gas purification box for testing. The exhaust gas component purification efficiency was calculated according to the initial concentration and the concentration at any time by Formula (1). However, the reduction of the exhaust gas concentration in the purification tank was composed of two parts, one of which was purified by the photocatalytic material and the other of which was extracted by the test instrument. Therefore, the process of determining which part was taken out by the instrument and which part was purified by materials was the key to ensuring the accurate calculation of photocatalytic efficiency. This article adopted the correction method to solve the problem of system error. The specific method was to use a large amount of blank test data to determine the correction value, which was the so-called blank test. The blank sample test used an exhaust gas purification test system. First, a sample was placed in the system, then it was tested again according to the purification process, and the data were recorded. After a large number of repeated tests, a large amount of data were obtained. The exhaust gas purification test was recorded every 10 min, so the blank test data were also recorded every 10 min. In this way, it could better reflect how the purification efficiency changed every 10 min. After a large number of blank sample tests, the final data are shown in Table 3. As can be seen from the table, the concentration of each component was reduced every 10 min with the passage of time. This was because the concentration of the gas in the environment box was decreasing, which also caused the concentration of the same volume of gas to be drawn down.

### 2.5. Analysis of Exhaust Gas Degradation Efficiency

According to the above experimental procedure in Section 2.3, the degradation efficiency of the g-C_3_N_4_/CeO_2_ composite that was prepared at different mass ratios could be calculated. The optimum ratio of g-C_3_N_4_ to CeO_2_ could be obtained. Moreover, the degradation efficiency of tail gas at the optimum ratio was further studied.

### 2.6. Microscopic Test

Studies have shown that CeO_2_ has a good visible light absorption capability and can degrade organic pollutants. The photogenerated electron-hole pair of g-C_3_N_4_ is easy to recombine when coupled with its low quantum efficiency and low catalytic performance, which limits its scale application in the field of degradation tail gas. Therefore, according to the band principle, g-C_3_N_4_/CeO_2_ composites were prepared. The newly formed composite material was a special heterojunction structure, and the structure and morphology of the composite material were studied by microscopic characterization methods such as XRD, FT-IR, UV-Vis, TG-DSC, SEM and XPS.

## 3. Results and Discussion

### 3.1. Degradation Principle of g-C_3_N_4_/CeO_2_

**Figure 2:**
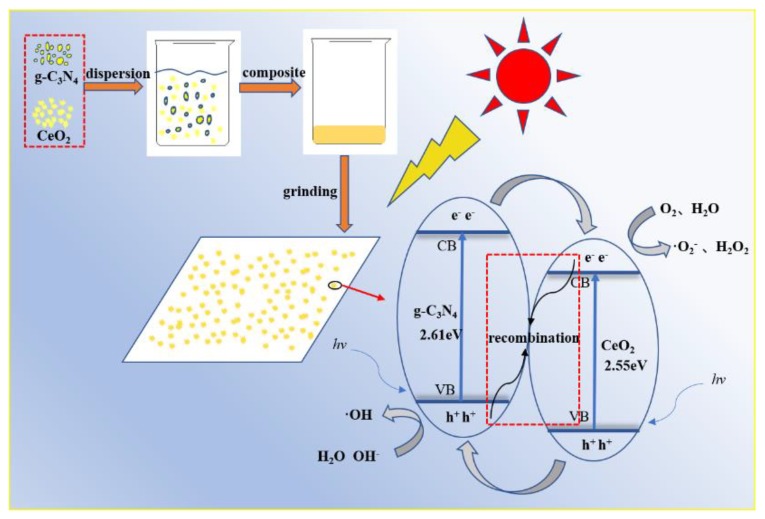
Principle of purifying exhaust gas with a g-C_3_N_4_/CeO_2_ composite material.

Figure 2 shows the principle of purifying exhaust gas by the g-C_3_N_4_/CeO_2_ composite material. The conduction band is the energy space formed by free electrons. The valence band usually refers to the highest energy band in a semiconductor or insulator that can be filled with electrons at 0 K. It can be seen from the Figure 2 that, due to the special relationship between the valence and conduction bands of the CeO_2_ material and the g-C_3_N_4_ material, a composite heterojunction photocatalytic material was formed. If the photon energy was higher than that of the photocatalyst absorption threshold when the sunlight irradiated g-C_3_N_4_/CeO_2_, the valence band electron (e^−^) of g-C_3_N_4_/CeO_2_ occurred inter-band transition. When an electron transitioned from the valence band to the conduction band, a corresponding hole (h^+^) was generated in the valence band. Then, a series of reactions occurred between the photo-generated electrons and the holes. For example, O_2_ was adsorbed on the surface of g-C_3_N_4_/CeO_2_ catalyst to form •$O_{2}^{-}$ by catching electrons, and the generated holes could oxidize water on the surface of g-C_3_N_4_/CeO_2_ to •OH. •$O_{2}^{-}$ and •OH were strong oxidizing groups that could oxidize most organic pollutants to CO_2_ and H_2_O. Since photogenerated electrons and holes were opposite charges, they would recombine through electrostatic forces, releasing or emitting energy to consume energy. The photo-generated carriers of g-C_3_N_4_ were easy to recombine. When the CeO_2_ material was added, the photo-generated electrons could be transferred from the conduction band of g-C_3_N_4_ to the conduction band of CeO_2_, and the photo-generated holes could be transferred from the valence band of CeO_2_ to the g-C_3_N_4_ valence band. Therefore, the g-C_3_N_4_/CeO_2_ photocatalytic material could effectively solve the problem of the low photo-generated carrier separation efficiency of the g-C_3_N_4_ material and could promote the dissociation of excitons, achieving the purpose of improving the photocatalytic activity of the g-C_3_N_4_ catalyst.

### 3.2. Degradation Efficiency of g-C_3_N_4_/CeO_2_

**Figure 3:**
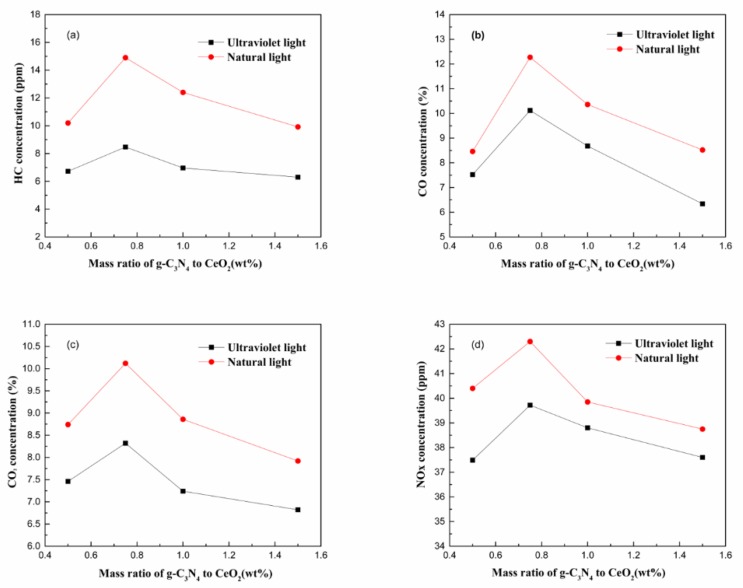
The purification effective change pattern at different g-C_3_N_4_ to CeO_2_ mass ratios for (**a**) HC, (**b**) CO, (**c**) CO_2_, and (**d**) NO_X_.

The degradation efficiencies of the binary composite that were obtained at different g-C_3_N_4_ to CeO_2_ mass ratios was tested by the method described in Section 2.3. The photocatalytic purification effective after error compensation is shown in Figure 3.

It can be seen from the four graphs in Figure 3 that the degradation efficiency of exhaust gas under natural light conditions was better than that under ultraviolet light, and in both natural and ultraviolet light conditions, the degradation efficiency of the four components in the automobile exhaust had a peak. When the mixing mass ratio of g-C_3_N_4_ to CeO_2_ was less than 0.75, the purification rate of each component increased continuously with the ratio increases. When the mixing mass ratio of g-C_3_N_4_ to CeO_2_ was greater than 0.75, the purification rate of each component was continuously reduced with the ratio increases. From the above results, it could be concluded that the optimum blend ratio of g-C_3_N_4_ to CeO_2_ was 0.75.

**Figure 4:**
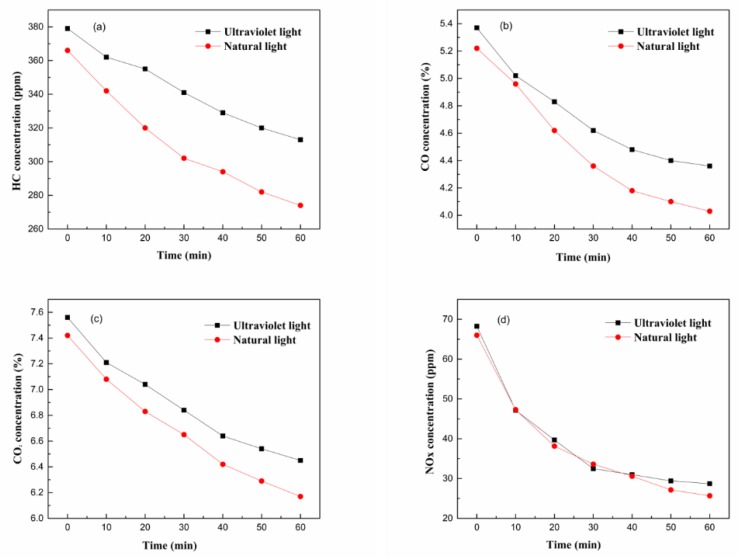
The change pattern of exhaust gas concentration (**a**) HC; (**b**) CO; (**c**) CO_2_; and (**d**) NO_X._

From the above analysis, when the mixing mass ratio of g-C_3_N_4_ to CeO_2_ was 0.75, the binary composites had the highest purification efficiency for the four components in the exhaust gas under both natural light and ultraviolet light conditions. However, it could not indicate how the exhaust gas concentration changed every 10 min over 60 min. Therefore, when the mass ratio of g-C_3_N_4_ to CeO_2_ was 0.75, the change trends of four components HC, CO, CO_2_ and NO_X_ were studied. The results that were obtained are shown in Figure 4.

It can be seen from Figure 4 that under ultraviolet light conditions, the degradation efficiencies of the g-C_3_N_4_/CeO_2_ composites for HC, CO, CO_2_ and NO_X_ in 60 min were 7.59%, 12.10%, 8.25% and 36.82%, respectively. Under visible light irradiation, the degradation efficiencies of HC, CO, CO_2_ and NO_X_ in the 60 min were 15.88%, 16.22%, 10.45% and 40.58%, respectively. The trends of HC, CO, CO_2_, and NO_X_ concentration patterns were concave curves, indicating that the degradation rate for exhaust gas in the initial stage was higher. With the extension of time, the purification and degradation rate of exhaust pollutants gradually decreased.

### 3.3. Micro Analysis

#### 3.3.1. UV-Vis Analysis

**Figure 5:**
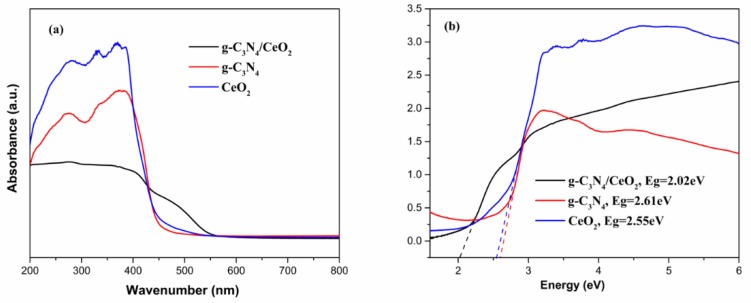
(**a**) UV-Vis DRS, (**b**) plots of (*αhν*)^1/2^ vs. photon energy of g-C_3_N_4_, CeO_2_, g-C_3_N_4_/CeO_2_ samples.

Figure 5a shows UV-Vis DRS spectra of g-C_3_N_4_, CeO_2_ and g-C_3_N_4_/CeO_2_. All the samples showed an adsorption edge in the visible light region. The g-C_3_N_4_/CeO_2_ material showed an adsorption edge of 550 nm, signifying its photocatalytic activity under visible-light irradiation and a significant blue-shift was observed for g-C_3_N_4_ and CeO_2_ [30]. Based on the electronic absorption spectra, the band gap energy could be obtained [31]. The calculated band gap value of the samples is shown in Figure 5b. The band gaps of g-C_3_N_4_, CeO_2_ and g-C_3_N_4_/CeO_2_ were 2.61, 2.55, and 2.02 eV, respectively. The g-C_3_N_4_/CeO_2_ binary composite showed a certain extent of red shift, corresponding to the high utilization of the visible light [32].

#### 3.3.2. FI-TR Analysis

**Figure 6:**
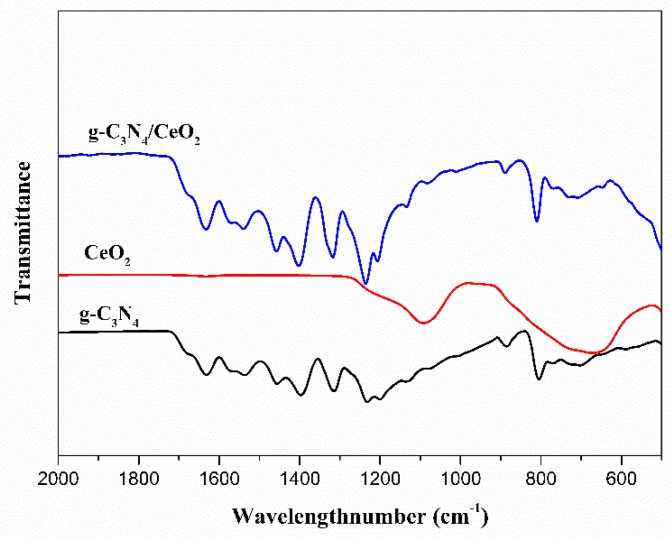
Infrared vibrational spectrum of the g-C_3_N_4_/CeO_2_ binary composite.

Figure 6 shows the infrared vibrational spectrum of the prepared g-C_3_N_4_/CeO_2_ binary composite at 2000–500 cm^−1^. As can be seen from Figure 6, there was a significant difference in the infrared spectra between the two materials between 500 and 1300 cm^−1^. Among them, the characteristic absorption peak in the range of 500–900 cm^−1^ was the stretching vibration absorption peak of Ce-O-Ce corresponding to the CeO_2_ material. The characteristic absorption peak in the range of 1250–1725 cm^−1^ was the typical stretching vibration absorption of the C–N heterocyclic ring corresponding to the g-C_3_N_4_ material. It was a typical 3-s-triazine structure out-of-plane bending vibration peak at 1280 and 1458 cm^−1^. This indicated that the sample was the binary composite of g-C_3_N_4_ and CeO_2_. In other ranges, the infrared spectra of the two materials were similar, probably due to the lower specific gravity of the CeO_2_ material, or possibly because the infrared absorption peak of the g-C_3_N_4_ material was stronger.

#### 3.3.3. XRD

**Figure 7:**
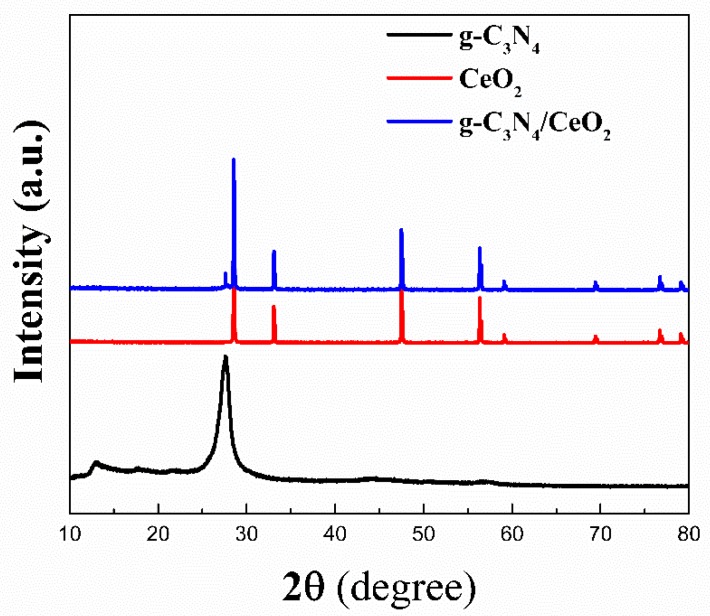
XRD pattern of g-C_3_N_4_/CeO_2_.

Figure 7 is an XRD pattern of the g-C_3_N_4_/CeO_2_ binary composite that was prepared by the optimum ratio obtained in Section 2.2. It can be seen from the figure that the peaks at the diffraction angles at 16.64° and 27.5° were characteristic peaks of g-C_3_N_4_. The diffraction peaks at 28.57°, 31.28°, 47.50°, 56.34°, 59.49°, 69.41°, 76.71° and 79.79° corresponded to the (111), (200), (220), (311), (222), (400), (331) and (420) crystal planes, respectively, of cubic fluorite-type CeO_2_. They all corresponded to the standard JCPDS NO.34-0394 card of CeO_2_ [23]. As can be seen from the figure, the mixed diffraction peaks of the two materials were relatively obvious, the peak width was narrow, and the crystallinity was good, so the prepared binary composite crystal form was better. Additionally, no other new crystal phase was found in the prepared binary composite material, indicating that the obtained sample was a composite of the g-C_3_N_4_ and CeO_2_ materials.

#### 3.3.4. TG-DSC

**Figure 8:**
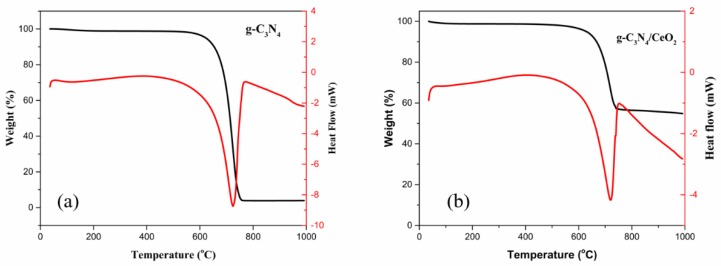
TG-DSC analysis of (**a**) g-C_3_N_4_ and (**b**) g-C_3_N_4_/CeO_2_.

Figure 8a is a TG-DSC analysis chart of g-C_3_N_4_. The TG and DSC diagrams of g-C_3_N_4_ could clearly reflect the following phase transition processes: When the temperature was raised from 0 to 609 °C, g-C_3_N_4_ absorbed heat slowly, while the TG data showed that the quality did not significantly change at this stage. This was because the melting point of g-C_3_N_4_ prepared in this work was about 609 °C, which was produced by endothermic melting. When the temperature was raised from 609 to 761 °C, the mass percentage of g-C_3_N_4_ sharply dropped, and the loss amount reached 92.07%. There was a strong exothermic peak at about 724 °C. This was because the temperature was too high, g-C_3_N_4_ was completely decomposed or sublimated, and the mass percentage at this time was zero.

Figure 8b shows a TG-DSC analysis chart of g-C_3_N_4_/CeO_2_. The TG and DSC diagrams of g-C_3_N_4_/CeO_2_ clearly reflect the following phase transition processes: From 0 to 609 °C, g-C_3_N_4_/CeO_2_ slowly absorbed heat, and the TG data showed that the quality did not significantly change at this stage. This was because the melting points of g-C_3_N_4_ and CeO_2_ were about 609 and 2400 °C. g-C_3_N_4_ was melted endothermically. When the temperature was raised from 609 to 761 °C, the mass percentage sharply dropped, and the mass loss reached 42.86%. There was a strong exothermic peak around 753 °C as well. This was because the temperature was too high, g-C_3_N_4_ was completely decomposed or sublimated, and the mass percentage at this time was 57.14%. It can be speculated that the remaining material was CeO_2_. It could be found from the mass loss percentage that the mixing mass ratio of g-C_3_N_4_ to CeO_2_ was 0.75. The right shift of the exothermic peak of the g-C_3_N_4_/CeO_2_ composite also showed that the two materials were connected together in the form of covalent bonds.

#### 3.3.5. SEM

**Figure 9:**
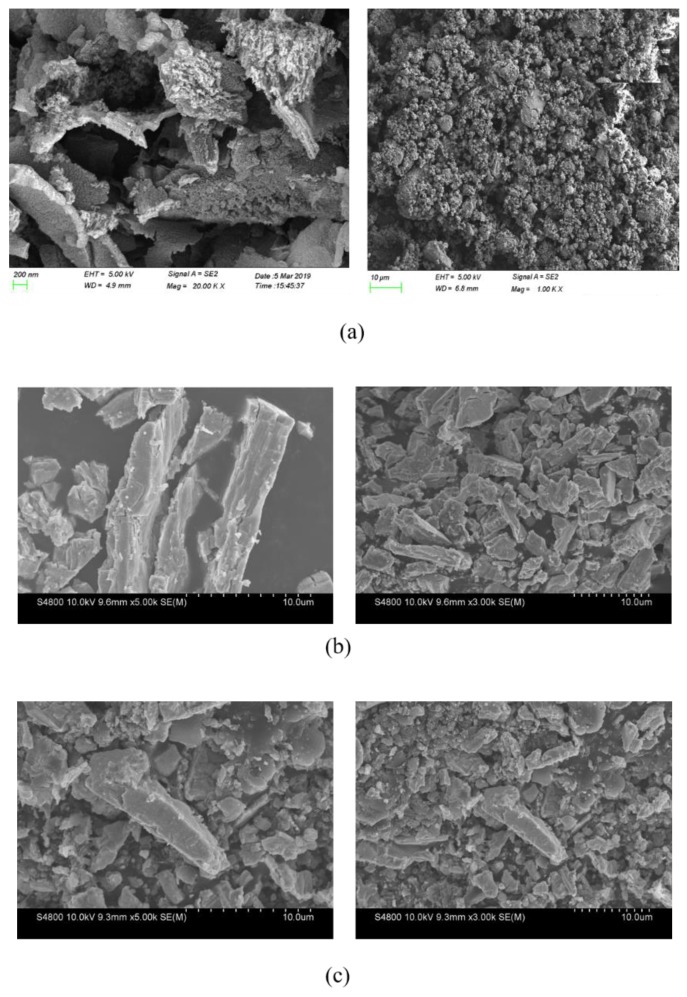
SEM images of (**a**) g-C_3_N_4_, (**b**) CeO_2_ and (**c**) g-C_3_N_4_/CeO_2_.

Figure 9 is the SEM images of g-C_3_N4 [33], CeO_2_, and g-C_3_N4/CeO_2_. As can be seen from the Figure 9a,b, g-C_3_N4 is granular and porous, and CeO_2_ is a rod-like structure. Figure 9c is the SEM image of g-C_3_N4/CeO_2_. It can be clearly seen from Figure 9 that layered g-C_3_N_4_ was attached to CeO_2_. This fully and directly shows that g-C_3_N_4_/CeO_2_ is a result of mutual doping.

#### 3.3.6. XPS

**Figure 10:**
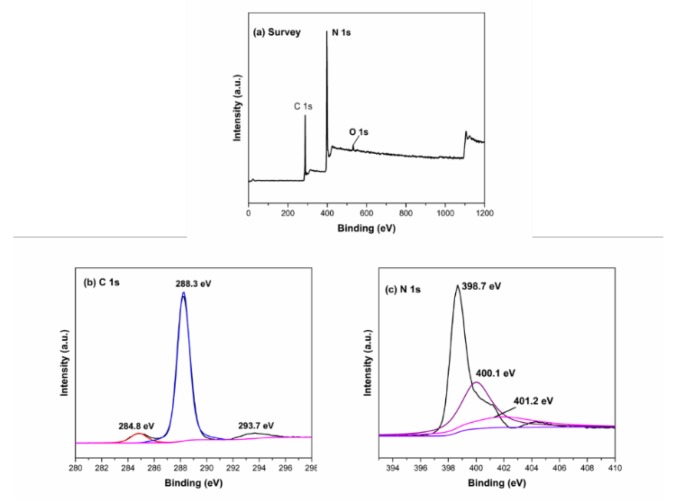
XPS spectrum of g-C_3_N_4_: (**a**) survey, (**b**) C 1s and (**c**) N 1s.

Figure 10 is the XPS spectrum of g-C_3_N_4_. As can be seen from Figure 10a, the sample contained three elements of C, N, and O. The O element may have been caused by the sample not being dried or the surface having H_2_O molecules. Figure 10b is the C 1s spectrum of g-C_3_N_4_. It can be seen from the figure that the peaks appeared at the binding energies of 284.8, 288.3, and 293.7 eV, which corresponded to the C=C bond in the graphite phase carbon nitride and the NC=N and C–NH_2_ bonds in the aromatic structure. Figure 10c is the N 1s spectrum of g-C_3_N_4_. It can be seen from the figure that peaks appeared at the binding energies of 398.7, 400.1, and 401.2 eV, respectively, corresponding to the CN = C bond in the triazine ring structure.

**Figure 11:**
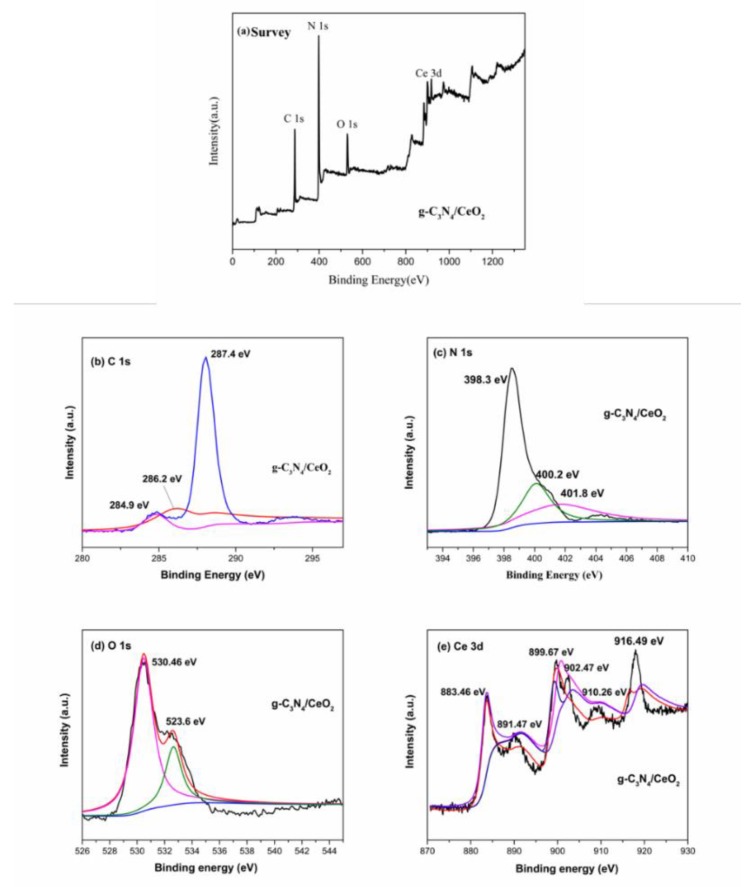
XPS spectrum of g-C_3_N_4_/CeO_2_: (**a**) survey, (**b**) C 1s, (**c**) N 1s, (**d**) O 1s and (**e**) Ce 3d.

Figure 11 is the XPS spectrum of g-C_3_N_4_/CeO_2_. Figure 11a is the full scan spectrum of the g-C_3_N_4_/CeO_2_ sample. It can be seen from the figure that only C, N, Ce and O were detected in the g-C_3_N_4_/CeO_2_ binary composite material. The prepared heterojunction composite was very pure, without other impurities. Figure 11b is the C 1s spectrum of the g-C_3_N_4_/CeO_2_ sample. It can be seen from the figure that the peaks appeared at the binding energies of 284.9, 286.2, and 287.4 eV, which corresponded to the C–C, C–NH_2_, and N=C–N_2_ bonds in the s-triazine ring, respectively. Figure 11c is the N 1s spectrum of the g-C_3_N_4_/CeO_2_ sample. It can be seen from the figure that the binding energy appeared peaks at 398.3, 400.2 and 401.8 eV. Among them, the main peak at the binding energy of 398.3 eV was an sp^2^ heterocyclic nitrogen atom in the C=N bond. The peak at a binding energy of 400.2 eV corresponded to the nitrogen atom in the N–(C)_3_ group. The corresponding peak at 401.8 eV was the nitrogen atom in the -NH_2_ bond. Figure 11d is the O 1s spectrum of the g-C_3_N_4_/CeO_2_ sample. It can be seen from the figure that the binding energy peaks appeared at 523.6 and 530.5 eV, which corresponded to the OH bond in the surface-attached water and Ce–O bond, respectively. Figure 11e is the Ce 3d spectrum of the g-C_3_N_4_/CeO_2_ sample. It can be seen from the figure that there were many peaks of Ce element. Through the analysis of the Gaussian–Lorentzian function, it is possible to conclude that the characteristic peaks of Ce^4+^ 3d^5/2^ and 3d^3/2^ were located at the binding energies of 883.5 and 902.5 eV. Moreover, the characteristic peaks of Ce^3+^ 3d^5/2^ and 3d^3/2^ were located at the binding energies of 891.5 and 910.3 eV. This indicated that Ce element mainly existed in the trivalent and tetravalent states in the material.

## 4. Conclusions

The main content of this work is that a g-C_3_N_4_/CeO_2_ heterostructure nanocomposite was prepared. The best mass ratio preparation of g-C_3_N_4_/CeO_2_ was obtained by indoor exhaust gas degradation. The degradation efficiency for HC, CO, CO_2_ and NO_X_ were obtained under natural and ultraviolet light irradiation. Moreover, XRD, FI-IR, UV-Vis, TG-DSC, SEM, and XPS were conducted to investigate the morphology and structure of g-C_3_N_4_/CeO_2_ heterostructure binary composite. The main conclusions are as follows:

(a) The exhaust gas purification efficiency of the g-C_3_N_4_/CeO_2_ composite under natural light irradiation was higher than that under ultraviolet light. The best mass ratio preparation of g-C_3_N_4_/CeO_2_ was 0.75.
(b) The degradation efficiencies of the g-C_3_N_4_/CeO_2_ composite for HC, CO, CO_2_ and NO_X_ in 60 min were 7.59%, 12.10%, 8.25% and 36.82%, respectively. Under natural light irradiation, the degradation efficiencies for HC, CO, CO_2_ and NO_X_ in the 60 min were 15.88%, 16.22%, 10.45% and 40.58%, respectively.
(c) The microstructure characterized the crystal structure and micro-morphology of g-C_3_N_4_/CeO_2_ composite. This indicated that g-C_3_N_4_/CeO_2_ heterostructure nanocomposite was successfully prepared.

## Figures and Tables

**Table 1:** Test equipment.

Device Name	Model	Factory
Alumina crucible	Φ100 mL	Shanghai Kesheng Ceramics Co., Ltd. (Shanghai, China)
Electronic balance	FA2004B	Shanghai Jingke Tianmei Scientific Instrument Co., Ltd. (Shanghai, China)
Muffle furnace	100 mL	Shanghai Pudong Physical Optical Instrument Factory (Shanghai, China)
Planetary ball mill	450 rpm	Changsha Miqi Equipment Co., Ltd. (Changsha, China)
Magnetic stirrer	0–2400 r/min	Changzhou Deke Instrument Manufacturing Co., Ltd. (Changzhou, China)
Electric centrifuge	0–4000 rpm	Jintan District Xicheng Xinrui Instrument Factory (Changzhou, China)

**Table 2:** Test raw materials.

Reagent Name	Model	Factory
Dicyandiamide	AR	Tianjin Fuchen Chemical Reagent Factory (Tianjin, China)
Cerium Oxide	AR	Tianjin Kemiou Chemical Reagent Co., Ltd. (Tianjin, China)

AR refers to analytical purity.

**Table 3:** Exhaust gas purification system error correction table.

Exhaust Gas Composition	0–10 min	10–20 min	20–30 min	30–40 min	40–50 min	50–60 min
HC (ppm)	10	6	8	6	6	5
CO (%)	0.1	0.06	0.06	0.06	0.06	0.06
CO_2_ (%)	0.1	0.1	0.1	0.1	0.08	0.08
NO_X_ (ppm)	8	5	6	2	1	1
